# CCN3 is a therapeutic target relating enhanced stemness and coagulation in hepatocellular carcinoma

**DOI:** 10.1038/s41598-017-14087-4

**Published:** 2017-10-23

**Authors:** Qingan Jia, Tongchun Xue, Qiangbo Zhang, Wei Cheng, Chun Zhang, Jingwei Ma, Yang Bu, Songning Yu, Qingguang Liu

**Affiliations:** 1 Department of Hepatobiliary Surgery, First Affiliated Hospital of Xi’an Jiaotong University, Xi’an, 710061 China; 2 Liver Cancer Institute, Zhongshan Hospital, Fudan University, Shanghai, 200032 China; 3 Department of General Surgery, Qilu Hospital, Shandong University, Jinan, 250012 China; 4 Department of Pharmacy and Medical Technology, Hanzhong Vocational and Technical College, Hanzhong, 723002 China; 5 Department of Hepatobiliary Surgery, General Hospital, Ningxia Medical University, Yinchuan, 750001 China

## Abstract

The general prognosis of patients with hepatocellular carcinoma (HCC) remains extremely dismal, due to the high frequency of metastasis. Since 2003, our research group has explored the gene expression profiles of metastasized HCC tissue samples and identified a significant upregulation of CCN3. However, the role and precise pathological function of CCN3 remains elusive. We showed that CCN3 is associated with the poor prognosis of patients with HCC, the malignant phenotype of HCC, and vascular thrombosis. We further evaluated the negative roles of CCN3 *in vitro* and *in vivo*, and identified osteopontin (OPN), and coagulation factors tissue factor (TF) and thrombin as the leading genes downstream of CCN3, that are positively associated with HCC cell stemness. We demonstrated that overexpressed CCN3 in HCC cells leads to enhanced survival and increased number of pulmonary metastases *in vivo*. The elevated levels of OPN and TF were associated with signal activation of nuclear factor κB (NFκB) and extracellular signal-regulated kinases (ERK). Our findings suggest CCN3 is a potential therapeutic target that would affect the upregulation of OPN and coagulation factors, which would lead to an enhanced stemness and blood coagulation microenvironment in HCC tissue.

## Introduction

Liver cancer is the second leading cause of cancer death worldwide, with about 50% of cases occurring in China. In particular, hepatocellular carcinoma (HCC) accounts for 80% of primary liver cancer cases worldwide^1^. Because HCC is typically diagnosed at an advanced stage, less than 30% of patients with HCC have the option of curative treatment, and the general prognosis of HCC patients remains extremely dismal. This unfavorable outcome is attributed to the high frequency of HCC metastasis and emphasizes the importance of exploring molecular signatures of metastasis and intervention strategies^2^. Since 2003, our research group has explored the expression profiles of metastasized HCC samples^3^. Our reanalysis of the cDNA microarray-based gene expression profile of HCC samples identified CCN3 (a matricellular protein that is encoded by the *NOV* gene in humans) as a significantly upregulated gene.

The expression levels of genes in the CCN family in HCC are closely correlated with certain biological characteristics and clinical features (e.g., venous invasion, cellular differentiation, and tumor recurrence), all of which are valuable in determining patient prognosis^4–6^. In particular, CCN3 proteins emerge as localized, multitasking signal integrators in the microenvironment and play an important role in modifying the cellular phenotype^7^. To date, only one study has described the enhanced expression of CCN3 in HCC samples compared with CCN3 levels in matched non-cancerous tissues. Nonetheless, both the precise physiological function and mechanism of action of CCN3 remain elusive^4^, and insight into the role of CCN3 in HCC is urgently needed for improvement of metastasis detection and development of targeted therapy.

Our findings in this study suggest that CCN3 could be a potential therapeutic target affecting the upregulation of OPN and activation of coagulation factors, which would then lead to an enhanced stemness and coagulation microenvironment in HCC tissue.

## Results

### Expression of CCN3 is positively associated with malignancy and poor prognosis

**Figure 1:**
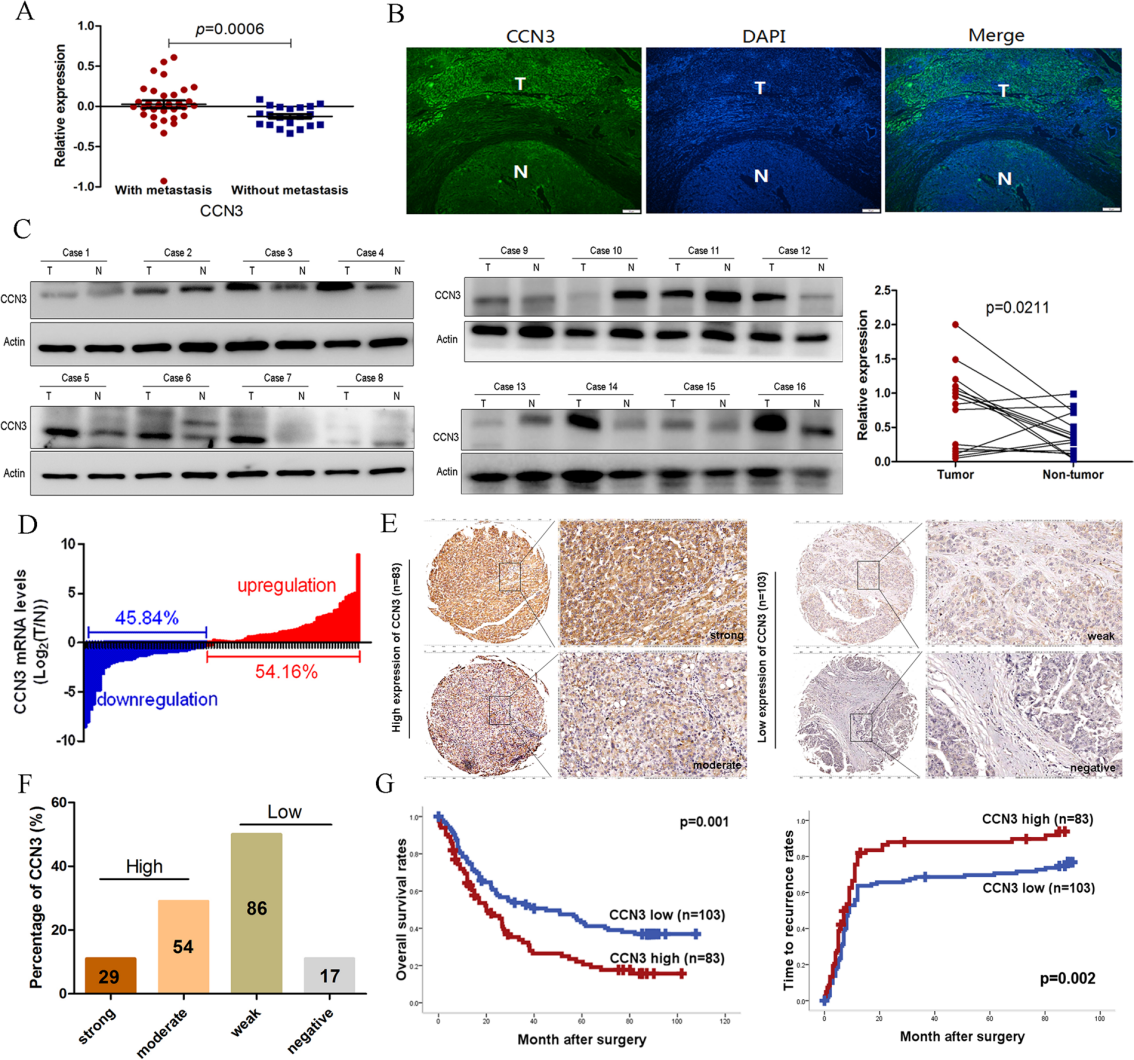
Expression of CCN3 is positively associated with malignancy and poor prognosis of HCC. (**A**) The upregulation of CCN3 identified in the gene profiles of metastatic HCC was compared by Student’s *t*-test. (**B**) Immunofluorescence showed higher CCN3 expression in HCC tissues. (**C**) The increased CCN3 expression levels in HCC tissues confirmed by immunoblot analysis were compared by Student’s *t*-test., and the gels/blots in each group were cropped from different gels. (**D**) An increased CCN3 expression level was observed in 54.16% of the HCC tissues compared with non-tumor liver tissues 45.84%. (**E**,**F**) On the basis of CCN3 expression levels, we sorted 186 HCC clinical samples in the validation cohort as high or low expression. (**G**) Patients in the CCN3-high group with significantly lower OS and higher TRR were performed by Kaplan-Meier analysis, and the values were generated by the Cox-Mantel log-rank test.

We reanalyzed the profile with HCC metastasis and found an upregulated expression of CCN3 in HCC samples (Fig. 1A). Immunofluorescence and immunoblotting assays revealed a higher expression of CCN3 in HCC tumor tissue samples than in non-tumor tissue samples (Fig. 1B and C). We also evaluated CCN3 mRNA expression levels in 48-paired HCC tumor tissue samples and in adjacent non-tumor liver tissue samples. We observed an increased CCN3 expression level in 54.16% (26/48) of the HCC samples compared to non-tumor liver tissue samples (Fig. 1D).

To illustrate the clinical role of CCN3 in HCC, we sorted 186 clinical samples in the validation cohort according to high or low CCN3 expression levels (Fig. 1E and F). The overall survival (OS) rates for CCN3-high patients at 1-, 3-, and 5- years (61.24, 33.45, and 22.17%, respectively) were significantly lower than those of CCN3-low patients (73.36, 51.26, and 42.78%, respectively). The 1-, 3-, and 5-year time to recurrence rates (TRR) of CCN3-high patients (76.64, 89.38, and 89.38%, respectively) were higher than those of CCN3-low patients (58.58, 67.13, and 69.98%, respectively). CCN3 expression was significantly associated with both OS and TRR. Patients in the CCN3-high group had significantly lower OS and significantly higher TRR than patients in the CCN3-low group (Fig. 1G). High expression of CCN3 was significantly correlated with vascular thrombosis and tumor dimension. No significant association was found between CCN3 expression level and the other clinical and pathological characteristics considered (Table 1).

**Table 1:** Association of CCN3 expression with the clinicopathologic characteristics of HCC patients (n = 186).

Variable	No. of Patients			Variable	No. of Patients		
	CCN3^low^	CCN3 ^high^	*P-*values		CCN3^low^	CCN3^high^	*P-*values
Age, y				Tumor dimension			
≥53	51	43	0.756	≥5 cm	13	22	**0.016**
<53	52	40		<5 cm	90	61	
Sex				No. of tumors			
Men	87	65	0.28	Multiple	4	2	0.693*
Women	16	18		single	99	81	
HBsAg				Vascular thrombosis			
Positive	97	81	0.320*	Yes	32	38	**0.0039**
Negative	6	2		No	71	45	
Cirrhosis				Tumor encapsulation			
Yes	94	75	0.832	Complete	51	43	0.858
No	9	8		None	52	40	
Serum, AFP				Tumor differentiation			
≥20	74	52	0.182	I–II	72	60	0.722
<20	29	31		III–IV	31	23	
Serum, ALT				OPN expression			
≥75	14	6	0.164	Low	51	25	**0.007**
<75	89	77		High	52	58	
TF expression							
Low	54	23	0.001				
High	49	60					

*Fisher’s exact tests, and Chi-square tests for all other analyses. For CCN3, TF and OPN median values were used as cut-off points for definition of subgroups (low expression and high expression groups). TF, tissue factor; OPN, osteopontin; AFP, alpha-fetoprotein; ALT, alanine aminotransferase; HBsAg, hepatitis B surface antigen; HCC, hepatocellular carcinoma.

We identified an association of tumor size, vascular invasion, and tumor number with both OS and TRR in HCC patients, using univariate analysis (Table 2). Using multivariate analysis models, we revealed that tumor size and tumor number are independent prognostic indicators of both OS and TRR in HCC patients. In contrast, vascular thrombosis was shown to be an independent prognostic indicator of only OS in HCC patients (Table 3).

**Table 2:** Univariate analyses of factors associated with OS and TTR of HCC patients (n = 186).

Variables	OS		TTR	
	Hazard ratio (95% CI)	*P-*values	Hazard ratio (95% CI)	*P-*values
Age, *y*, ≥53 vs <53	0.354(0.790–2.253)	0.412	1.048(0.615–1.502)	0.534
Sex, male vs female	0.917(0.480–1.720)	0.788	1.216(0.657–2.252)	0.533
HBsAg, positive *vs* negative	0.812 (0.338–1.641)	0.560	1.282(0.367–2.518)	0.818
Cirrhosis, no vs yes	1.052(0.595–1.859)	0.862	1.095(0.589–1.681)	0.886
AFP, *ng/mL*, ≥20 vs <20	1.461(0.816–2.642)	0.166	1.165(0.695–1.883)	0.490
ALT	0.864(0.535–2.193)	0.965	0.767(0.431–1.755)	0.840
Tumor size	2.915(1.667–5.312)	**0.006**	2.382(1.491–4.814)	**0.012**
No. of tumors	4.823(2.612–10.127)	**0.001**	6.194(2.825–12.256)	**0.002**
Vascular invasion, yes *vs* no	2.368(1.415–4.329)	**0.001**	1.821(1.199–2.972)	**0.020**
Tumor encapsulation	1.520(0.931–2.482)	0.086	1.602(1.261–2.418)	0.051
Tumor differentiation	1.326(0.716–2.308)	0.136	1.315(0.919–2.511)	0.197
CCN3, high vs low	1.391(0.912–1.998)	**0.001**	1.818(1.312–2.810)	**0.002**

Univariate analysis, Cox proportional hazards regression model. AFP, alpha-fetoprotein; ALT, alanine aminotransferase; CI, confidence interval; HBsAg, hepatitis B surface antigen; HCC, hepatocellular carcinoma; OS, overall survival; TTR, time to recurrence.

**Table 3:** Multivariate analyses of factors associated with OS and TTR of HCC patients (n = 186).

Variables	OS		TTR	
	Hazard ratio (95% CI)	*P-*value	Hazard ratio (95% CI)	*P-*value
Tumor size	1.582(1.241–2.185)	**0.001**	1.316(1.248–1.672)	**0.019**
No. of tumors	4.221(2.106–10.324)	**0.002**	5.042(2.412–11.249)	**0.013**
Vascular invasion, yes vs no	2.312(1.499–3.714)	**0.012**	1.511(0.972–2.492)	0.098
CCN3, high vs low	2.222(1.161–3.255)	**0.002**	2.812(1.297–4.131)	**0.008**

Multivariate analysis, Cox proportional hazards regression model. For CCN3 median values were used as cut-off points for definition of subgroups (low expression and high expression groups). OS, overall survival; TTR, time to recurrence.

### Upregulation of CCN3 is related to enhanced stemness

**Figure 2:**
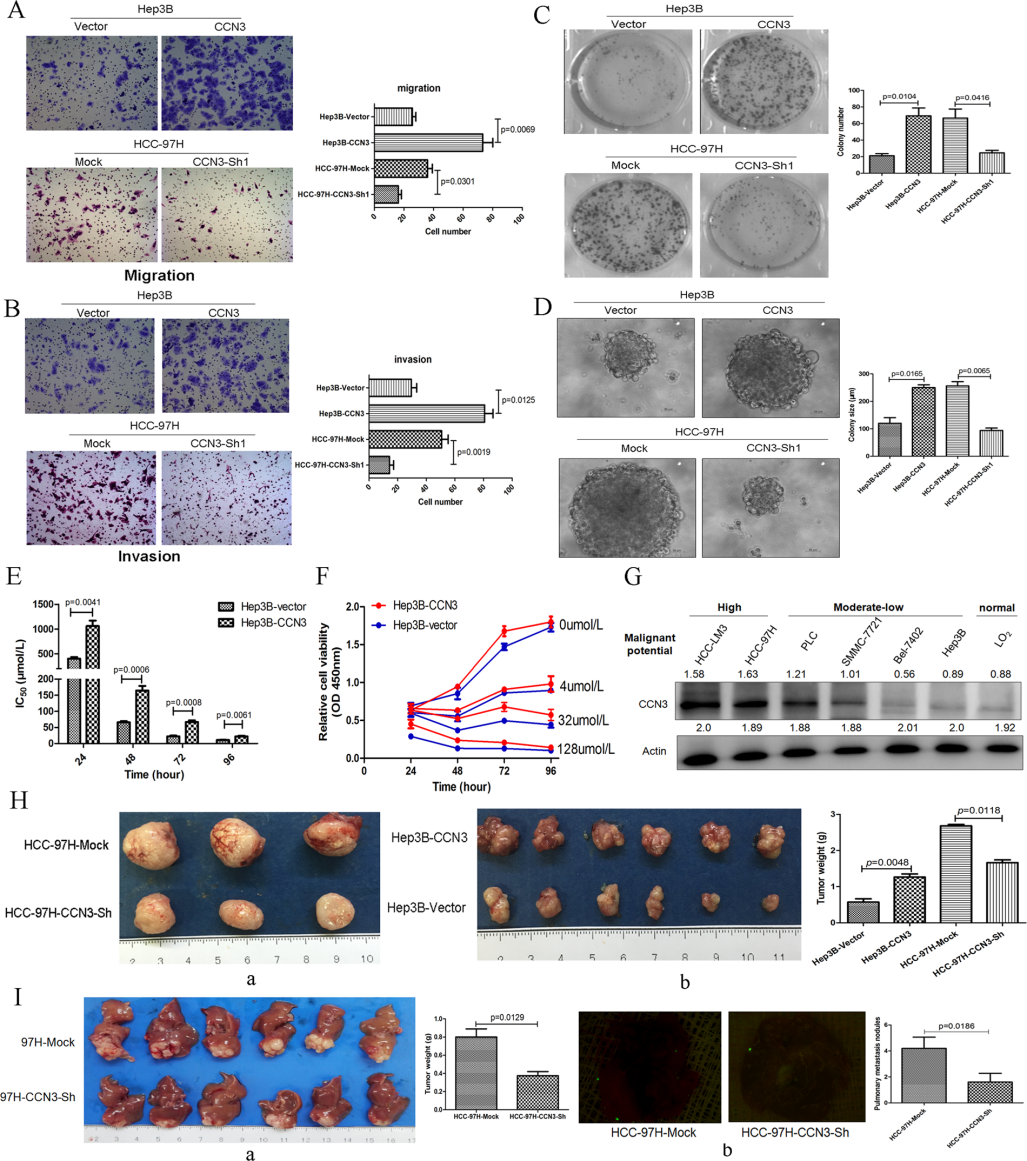
Upregulation of CCN3 is correlated to the enhancement of the malignant phenotype in HCC. The cell migration (**A**) and invasion (**B**) assays demonstrated that the number of cells crossing the basement membrane was higher for Hep3B-CCN3 cells than for HCC-97H cells, and was inhibited after downregulation of CCN3 in HCC-97H. Overexpression of CCN3 in Hep3B cells also enabled enhanced ability of proliferation (**C**), and sphere formation (**D**), which were inhibited after downregulation of CCN3 in HCC-97H. (**E**) Upregulation of CCN3 significantly enhanced oxaliplatin resistance with an increased IC_50_ after treatment with oxaliplatin for 24-96 h. (**F**) The stronger resistance in HCC was confirmed with overexpressed CCN3 after treatment with oxaliplatin in gradient concentrations (0-128 μmol/L). (**G**) High expression of CCN3 was found in HCC cell lines with high malignant potential, and the gels/blots in each group were cropped from different gels. (**H**) Diminished subcutaneous tumor size in nude mouse models was found in the HCC-97H-CCN3-sh group (n = 3, a), whereas enhanced tumor growth with increased subcutaneous tumor size was found in the Hep3B-CCN3 group (n = 6, b). (**I**) The weight of the orthotopic xenografts (a) and number of pulmonary metastasis nodules (b) were lower in the HCC-97H-CCN3-sh group than in the HCC-97H-mock group (n = 6). Quantitative differences in the data cell number, colony size and number, tumor weight, and pulmonary metastasis nodules were all evaluated by Student’s *t*-test.

The upregulation of CCN3 significantly enhanced the ability of Hep3B cells with regard to migration, invasion, proliferation, and sphere formation. HCC-97H cells transfected with CCN3-shRNA exhibited inhibited abilities for malignant phenotype mentioned above (Fig. 2A–D). Moreover, the upregulation of CCN3 significantly enhanced oxaliplatin resistance, which exhibited an increased IC_50_ of HCC cells to oxaliplatin (Fig. 2E). The stronger proliferation ability was also found in HCC with the upregulation of CCN3 after treatment with oxaliplatin (Fig. 2F). The HCC cell line with different metastatic potential were assessed and used in our previous study^8,9^. In this study, CCN3 levels were significantly increased in HCC cell lines with high metastatic potential (HCC-LM3 and HCC-97H), whereas relatively low CCN3 levels were detected in HCC cell lines with moderate-low metastatic potential (PLC, SMMC-7721, Bel-7402, and Hep3B) and in human liver cells (L02) (Fig. 2G).

**Figure 3:**
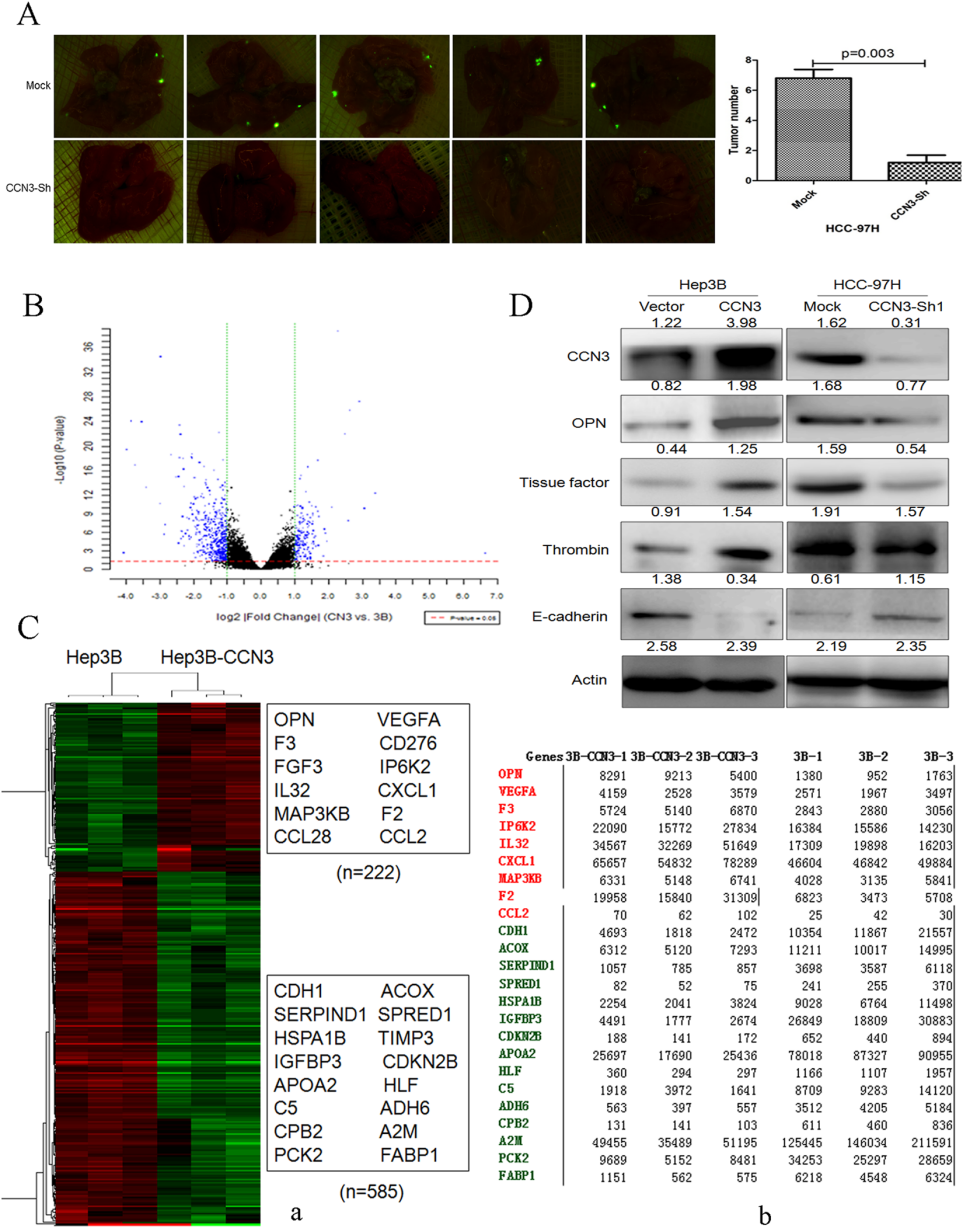
Downregulation of CCN3 in HCC is associated with decreased pulmonary metastasis nodules, and gene expression profiles are significantly changed after CCN3 overexpression (**A**) HCC cells with downregulated CCN3 exhibited reduced pulmonary metastasis nodules were compared by Student’s *t*-test. (**B**) Each point is a detected gene probe which showing the distribution of differential gene probes in the grouping. And expression levels of 807 genes, including 222 upregulated and 585 downregulated genes, were more than two-fold different between Hep3B-vector and Hep3B-CCN3 cells. (**C**) Gene expression profiles were evaluated by Gene Ontology (a). Expression levels of OPN, TF, thrombin, and CDH1 were significantly changed (b). (**D**) Immunoblotting was used to verify the upregulation of OPN, TF, and thrombin, and the downregulation of E-cadherin in HCC with overexpressed CCN3, and the gels/blots in each group were cropped from different gels and different fields. The downregulation of OPN, TF, and thrombin, and the upregulation of E-cadherin in HCC cell lines expressing shCCN3.

In nude mouse models, a diminished subcutaneous tumor growth was found in mice injected with HCC-97H-CCN3-sh cells. In contrast, the overexpression of CCN3 through lentiviral infection of Hep3B cells in mice led to enhanced subcutaneous tumor growth (Fig. 2H). Furthermore, the tumor weight of the orthotopic xenografts and number of pulmonary metastasis nodules were lower in the HCC-97H-CCN3-sh group than in the Mock group (Fig. 2I). HCC cells with downregulated CCN3, from male BALB/c nu/nu mice injected through the tail vein, exhibited decreased pulmonary metastasis (Fig. 3A).

### CCN3 overexpression alters gene expression profiles for OPN and coagulation factors

We compared the gene expression profiles of Hep3B-vector and Hep3B-CCN3 (2, 8264 genes). The expression of 807 genes, including 222 upregulated and 585 downregulated genes, were more than two-fold different between Hep3B-vector and Hep3B-CCN2 cells (Fig. 3B). The 807 differentially expressed genes in HCC with CCN3 overexpression were evaluated by Gene Ontology (GO) and pathway enrichment. Eleven genes related to complement and coagulation cascades were significantly changed, including TF and thrombin, which are closely related to hypercoagulable microenvironments (Fig. 3C).

Interestingly, *spp1* (secreted phosphoprotein 1), which encodes OPN and plays a crucial role in HCC stemness^3,10,11^, was significantly upregulated in HCC with overexpressed CCN3. On the other hand, *CDH1*, which encodes E-cadherin and mediates the epithelial phenotype in tumor cells^12^, was significantly downregulated in HCC with overexpressed CCN3. The downregulation of E-cadherin and upregulation of OPN, TF, and thrombin in HCC with overexpressed CCN3 were confirmed by immunoblotting (Fig. 3D).

### OPN and TF are positively associated with malignancy, especially vascular thrombosis

**Figure 4:**
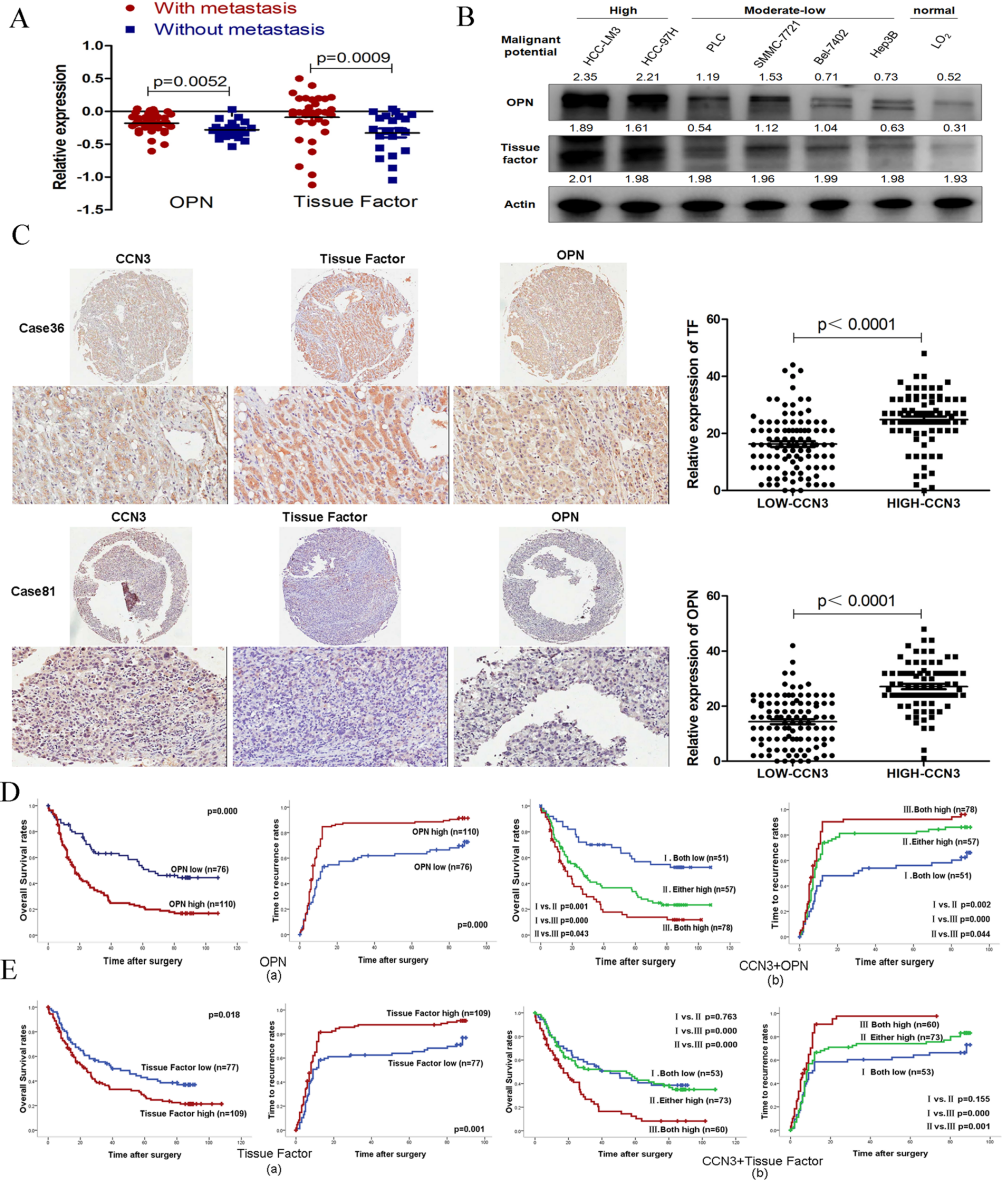
The expression of OPN and TF are positively associated with malignancy and poor prognosis of HCC patients. (**A**) Gene expression profiles of HCC samples with metastasis were reanalyzed, and the upregulation of OPN and TF in patients with metastasis was compared by Student’s *t*-test. (**B**) High expressions of OPN, and TF are found in HCC cell lines with high metastatic potential, and the gels/blots in each group were cropped from different gels. (**C**) A positive relationship among the expression of CCN3, TF, and OPN, in HCC was confirmed by immunohistochemistry, and the upregulation of TF, and OPN in patients with high expression of CCN3 was compared by Student’s *t*-test (**D**) Patients in the OPN-high group displayed significantly lower OS and higher TRR when combined with CCN3 (a). Patients with low levels of both CCN3 and OPN had the highest OS and lowest TRR (b). (**E**) Patients in the TF-high group displayed significantly lower OS and higher TRR when combined with CCN3 (a). Patients with low expression levels of both/or CCN3 and TF had the higher OS and lower TRR (b). Kaplan-Meier analysis was performed to compare TRR and OS between patients in different groups and statistic, and the values were generated by the Cox-Mantel log-rank test.

We reanalyzed the expression profiles of HCC samples with and without intrahepatic metastasis^3^ and identified the upregulation of OPN and TF in HCC with similar trends in intrahepatic metastasis as previously reported (Fig. 4A). Then, we investigated the roles of OPN and TF in seven HCC cell lines with different malignant phenotypes. OPN and TF expressions were significantly increased in HCC cell lines with high metastatic potential, whereas relatively low OPN and TF levels were detected in HCC cell lines with low metastatic potential and in human liver cells (Fig. 4B). To illustrate the roles of OPN and TF in HCC, we used tissue microarrays with 186 clinical samples and found a positive correlation among CCN3, OPN, and TF (Fig. 4C). Expression of OPN and TF were also significantly associated with both OS and TRR. The patients in the OPN-high group had significantly lower OS and higher TRR than patients in the OPN-low group (Fig. 4D). The patients in the TF-high group also had significantly lower OS and higher TRR than patients in the TF-low group (Fig. 4E).

Next, we classified the patients into three subgroups based on OPN and CCN3 expression levels. Group I had low expression levels of both CCN3 and OPN, Group II had high expression of either OPN or CCN3, and Group III had high expression of both CCN3 and OPN. Group I had the best prognosis because the OS rate of the patients was significantly higher than that of patients in Groups II and III. Also, the TRR in Group I was lower than those of Groups II and III. The OS was significantly higher and the TRR was significantly lower in Group I than in Group III (Fig. 4D). Based on TF and CCN3 expression levels, the patients were classified again into three subgroups. The patients in Group I (low expression levels of both CCN3 and TF) or Group II (high expression of either TF or CCN3) had better prognosis; their OS rate was significantly higher than that of patients in Group III (high expression of both TF and CCN3), and their TRR was significantly lower than that of patients in Group III; Between Group I and Group II, there was no significant different on the OS rate and TRR rate (Fig. 4E).

Furthermore, Cox regression analysis revealed a significant association of OPN-high expression in tumor tissue with tumor dimension and vascular invasion of HCC. A significant correlation between TF-high expression in tumor tissue and vascular invasion of HCC was also found (Table 4).

**Table 4:** Correlations among OPN, TF, and clinicopathology features in 186 patients with HCC.

Variable	No. of Patient			No. of Patient		
	OPN^low^	OPN^high^	*P-*value	TF^low^	TF^high^	*P-*value
**Age, y**						
≥53	37	57	0.674	42	52	0.358
<53	39	53		35	57	
**Sex**						
Men	65	87	0.264	62	90	0.722
Women	11	23		15	19	
**HBsAg**						
Positive	70	108	0.065*	74	104	0.819*
Negative	6	2		3	5	
**Cirrhosis**						
Yes	71	98	0.314	70	99	0.984
No	5	12		7	10	
**Serum, AFP**						
≥20	50	76	0.636	51	75	0.712
<20	26	34		26	34	
**No. of tumors**						
Multiple	3	3	0.643*	2	4	0.515*
Single	73	107		75	105	
**Tumor dimension**						
≥5 cm	13	22	0.62	13	22	0.571
<5 cm	63	88		64	87	
**Vascular thrombosis**						
Yes	18	52	**0.001**	20	50	**0.006**
No	58	58		57	59	
**Tumor encapsulation**						
Complete	36	58	0.472	40	54	0.746
None	40	52		37	55	
**Tumor differentiation**						
I–II	63	69	**0.003**	53	79	0.59
III–IV	13	41		24	30	

*Fisher’s exact tests, and Chi-square tests for all other analyses. For OPN and TF median values were used as cut-off points for definition of subgroups (low expression and high expression groups). TF, tissue factor; OPN, osteopontin; AFP, alpha-fetoprotein; HBsAg, hepatitis B surface antigen; HCC, hepatocellular carcinoma.

### Elevated levels of OPN, TF, and stemness-associated markers are associated with NFκB and ERK signaling activation after CCN3 overexpression

**Figure 5:**
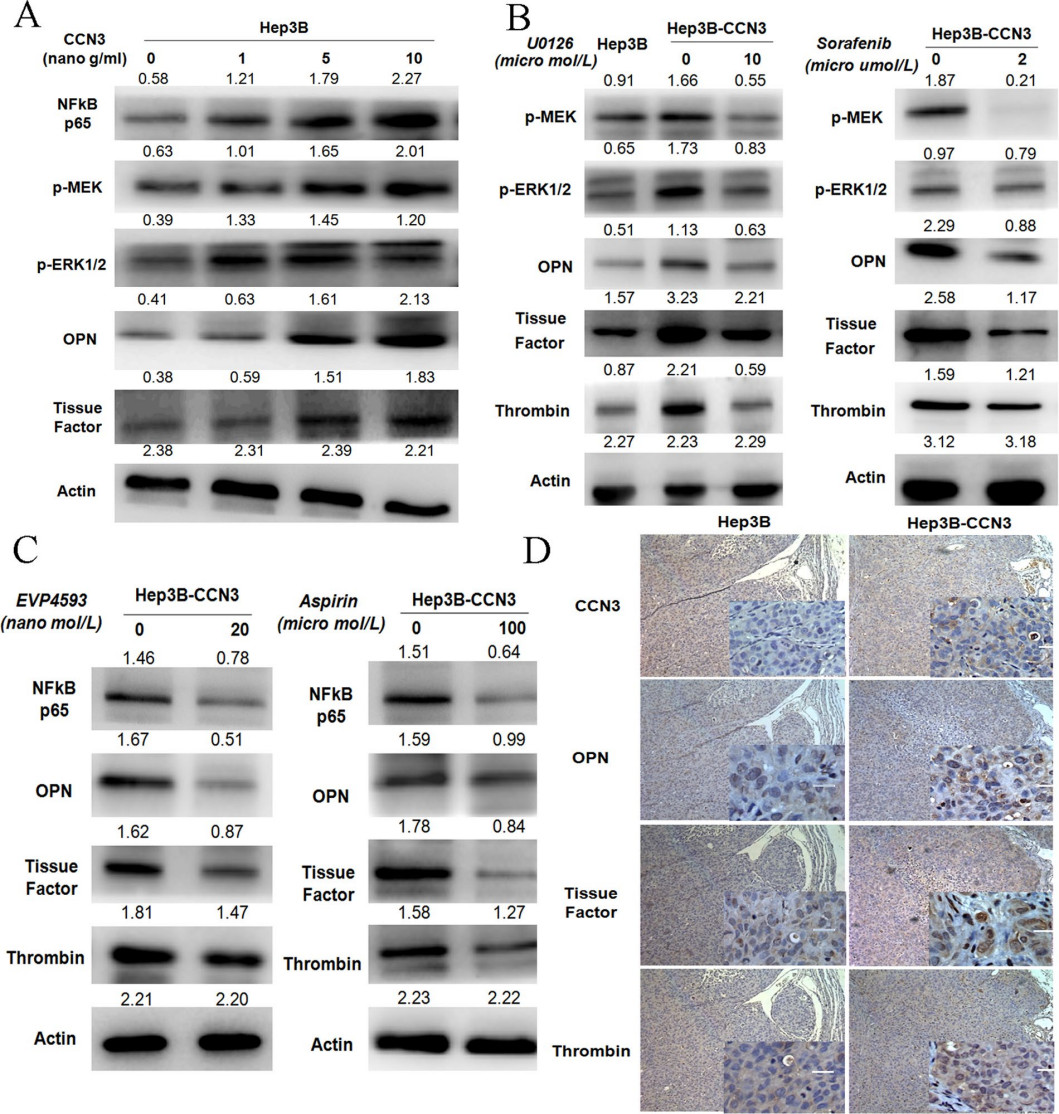
Elevated levels of OPN and TF are associated with NFκB and ERK signaling activation. (**A**) Hep3B cells treated with recombinant CCN3 in a dose-dependent manner exhibited activation of ERK and NFκB signaling with concomitant upregulation of OPN, TF, and thrombin, and the gels/blots in each group were cropped from different gels and different fields. (**B**) Hep3B-CCN3 cells treated with ERK signaling inhibitor sorafenib or U0126 exhibited a significant downregulation of OPN, TF, and thrombin, and the gels/blots in each group were cropped from different gels and different fields. (**C**) Hep3B-CCN3 cells treated with NFκB inhibitor EVP4593 or Aspirin exhibited a significant down regulation of OPN, TF, and thrombin, and the gels/blots in each group were cropped from different gels and different fields. (**D**) Immunohistochemical staining confirmed the significantly increased expression of OPN, TF, and thrombin in Hep3B-CCN3 subcutaneous tumors.

We overexpressed CCN3 in Hep3B cells and found this led to activated ERK and NFκB signaling pathways and upregulation of OPN, TF, and thrombin. To validate this effect, we treated HCC cells with recombinant CCN3 in a dose-dependent manner (0–10 micro g/mL) and confirmed the activation of ERK and NFκB signaling pathways with concomitant upregulation of OPN, TF, and thrombin (Fig. 5A). On the other hand, Hep3B-CCN3 cells treated with sorafenib (2 micro mol/L) or MEK1/2 inhibitor U0126 (10 micro mol/L) showed an enhanced inhibition of ERK signaling and downregulation of OPN, TF, and thrombin (Fig. 5B). Furthermore, NFκB signaling was inhibited with concomitant downregulation of OPN and TF after treatment with NFκB inhibitor EVP4593 or Aspirin (Fig. 5C). The enhanced expressions of OPN, TF, and thrombin after CCN3 overexpression were confirmed in Hep3B subcutaneous tumors by immunohistochemical staining (Fig. 5D).

## Discussion

Although progress has been made in clinical treatments for HCC, the general prognosis of patients with HCC remains extremely poor because of the high frequency of HCC metastasis^1^. Assessing the general condition of HCC patients, such as liver function, tumor stage, and vascular thrombosis, is useful in predicting HCC metastasis and the prognosis of patients. However, prediction of clinical outcomes is not always exact. Therefore, insights into the molecular signatures of HCC are urgently needed to improve metastasis prediction and targeted therapy.

CCN3 is one of a six-member family of cysteine-rich regulatory proteins found in humans. These proteins have emerged as localized multitasking signal integrators in the inflammatory microenvironment. Full-length CCN3 comprises a secretory signal peptide followed by four structural domains; namely, insulin-like growth factor binding proteins (IGFBP), von Willebrand factor type C repeat (VWC), thrombospondin type I repeat (TSP-1), and carboxy-terminal domain (CT)^7^. Recently, interest in CCN3 has emerged for cancer research because of the protein’s central roles in cell regulation. For instance, in osteosarcoma, high expression of CCN3 is significantly correlated with poor prognosis, and CCN3 may increase cell motility and MMP-13 expression through the integrin-dependent pathway^13^. In prostate cancer, CCN3 is a potential therapeutic target for prevention of bone metastasis via inhibition of the FAK/Akt/p38/NF-κB signaling pathway^14^. Since αvβ3 integrin is the receptor for CCN3, it is interesting to note that patients with high expression of αvβ3 also had significantly shorter survival^15^. However, the precise physiological function and mechanism of action of CCN3 in HCC malignancy remain vague^4^. In this study, we confirmed the prognostic utility and therapeutic value of CCN3 in HCC. Furthermore, when CCN3 was overexpressed, we found significant alterations in gene expression profiles, including the leading upregulation of OPN and coagulation factors TF and thrombin.

OPN is a matricellular protein that propagates cellular signals and promotes induction of cell adhesion, chemotaxis, extracellular matrix degradation, and other stemness-related characteristics^16^. Our research group has been studying OPN for 14 years to evaluate its prognostic and therapeutic value in HCC^3,8,10,11,17–19^. In the present study, we confirmed that high OPN expression is significantly correlated with tumor dimension and vascular thrombosis and that it has a positive association with CCN3. We found that patients with high expression levels of both CCN3 and OPN have the poorest prognosis, and CCN3 may be advantageous for the management of HCC metastasis because CCN3 correlates with OPN regulation and activation of the ERK and NFκB signaling pathways.

The hemostatic system is often subverted in patients with cancer, which results in life-threatening vascular thrombotic events^20^. Interestingly, the first manifestation of various tumors may present as hyperactivation of the coagulation system and thrombosis^21^. The expression and activity of cancer-derived TF is a major molecular driver of cancer-associated coagulopathy and thromboembolic disorders^22^. Paracrine TF converts the zymogen coagulation factor VII (FVII) to its activated state, which potentiates its catalytic efficiency in converting factor X (FX) to activated factor X (FXa), and finally activates factor IX (FIX) to FIXa to ultimately promote thrombin generation, fibrin formation, and platelet activation^22^. The highest levels of TF expression have been reported in cancers strongly associated with a high incidence of thrombotic events^23^. In this study, we demonstrated that a significant increase of TF expression in HCC leads to vascular thrombosis with short OS and high TRR in HCC patients. Expression of thrombin, a potential therapeutic target of HCC^8^, was also found to be associated with the expression of TF and CCN3 in this study. Our findings suggest that CCN3, acting as an upstream gene, could upregulate coagulation factors TF and thrombin, which are in turn correlated with ERK and NFκB signaling activation. On the other hand, downregulation of CCN3 or inhibition of ERK or NFκB signaling could significantly downregulate TF and thrombin.

In conclusion, we have provided evidence of CCN3 as a therapeutic target that relates enhanced stemness and coagulation in HCC. Cancer progression is profoundly influenced by factors that cause blood coagulation, particularly coagulation initiator TF. We showed that the enhanced coagulation induced by CCN3 might lead to formation of pulmonary metastasis and the poor prognosis of patients with HCC. And the possible association between the coagulation mechanism and metastasis may involve cluster-promotion ability of tumor cells, which ensures cell survival from anoikis and immune attack^24^. However, the relationship between tumor cells with differential procoagulant potential and the number of tumor clusters is vague. Further studies in our group will focus on exploring an association between HCC with overexpressed CCN3 and the number of tumor clusters in the circulation to find means for tumor inhibition via anticoagulants in tumor metastasis.

## Materials and Methods

### Patients and Follow-Up

Tissue samples were obtained from patients who had undergone curative liver resection (defined as complete resection of tumor nodules, leaving tumor margins free of cancer according to histologic examination) at the Liver Cancer Institute and Zhongshan Hospital of Fudan University between January 2004 and December 2006. Informed written consent was obtained from each participant. The clinical parts of the study were approved by the Research Ethics Committee of Fudan University and conducted according to the ethical guidelines outlined in the Declaration of Helsinki for use of human tissue and subjects. A total of 234 paired tissue specimens were obtained; 48 paired HCC samples were used for real-time polymerase chain reaction (PCR) analysis and 186 paired HCC samples were used for immunohistochemistry analysis. Histopathologic diagnosis was performed according to the World Health Organization criteria.

Patients were followed up after surgical treatment, as previously described, until December 2013^25^. The median follow-up period was 63 months (range, 0–110 months). Sixteen patients were followed up for immunobloting analysis. The clinicopathologic characteristics of all HCC patients in this study are provided in Table 1.

### Cell Lines and Animals

High metastatic potential human HCC cell lines used in the study included HCC-LM3 and HCC-97H (established at Fudan University)^26^. These cell lines were authenticated by Biosyn, Inc. in a 2010 study that used DNA profiling of short, tandem repeat markers. Low metastatic potential human HCC cell lines used in the study included SMMC-7721 (established at Second Military Medical University, China) and PLC, Bel7402, and Hep3B (American Type Culture Collection, Rockville, MD, USA). We also used human liver cell line LO2 (Chinese Academy of Science, Beijing, China). These cell lines were conserved and supplied by the Liver Cancer Institute at the time of our study initiation in 2010. All cells were maintained in Dulbecco’s Modified Eagle’s Medium (DMEM; GIBCO, Grand Island, NY, USA) supplemented with 10% fetal bovine serum (FBS; GIBCO) at 37 °C in a humidified incubator with 5% CO_2_. Cells were routinely screened for the presence of mycoplasma (Mycoplasma Detection Kit, Roche Diagnostics, Indianapolis, IN, USA).

Male BALB/c nu/nu mice (aged 4–6 weeks and weighing approximately 20 g) were obtained from the Chinese Academy of Science (SLRC, Shanghai, China) and maintained under standard pathogen-free conditions. Tumors were produced by administering subcutaneous injections of Hep3B-CCN3 and Hep3B-vector cells or HCC-97H-CCN3-sh and HCC-97H-mock cells into the upper left flank region of the mice. Orthotopic xenografts of mice were generated using subcutaneous tissues from mice injected with HCC-97H-CCN3-sh and HCC-97H-mock cells. The subcutaneous tumors were removed respectively, randomly selected tumor tissues of one mouse from each group and minced into equal size of 2 mm^3^, and randomly transplanted into the livers of mice. Five weeks later, orthotopic xenografts were measured and performed as described in previous publication^27^. Pulmonary metastasis was evaluated by fluorescence microscopy. HCC-97H-CCN3-sh and HCC-97H-mock cells were also injected into male BALB/c nu/nu mice through the tail vein. Animals were sacrificed five weeks after injections, and pulmonary metastatic nodules were evaluated by fluorescence microscopy. The study protocol was approved by the Shanghai Medical Experimental Animal Care Commission, and all methods were performed in accordance with the relevant guidelines.

### Cell Proliferation, Migration, Matrigel^®^ Invasion, and Sphere Formation Ability Assays

In cell proliferation assays, Hep3B-CCN3 and Hep3B-vector cells were cultured in 96-well plates (4 × 10^3^ cells/well). Cells were exposed to increasing concentrations of oxaliplatin (0, 0.5, 1, 2, 4, 8, 16, 32, 64, and 128 μmol/L) for 24, 48, 72, and 96 h. The relative number of cells was calculated using the Cell Counting Kit-8 (Dojindo, Kumamoto, Japan). Results were expressed as the absorbance of each well at 450 nm (OD 450). Invasion and migration of Hep3B-CCN3 and Hep3B-vector cells, HCC-97H-CCN3-sh and HCC-97H-mock cells were assessed by transwell assays using Boyden chambers (Corning, Tewksbury, MA, USA). Low adhesion and high adhesion 6-well plates were used for Colony formation assays, and detailed information was performed as previously described^28,29^.

### DNA Microarray Analysis

Changes in gene expression were evaluated by DNA microarray. Total RNA was extracted from Hep3B-CCN3 and Hep3B-vector cells. Isolations and microarray analyses were performed in triplicate using the Human OneArray® probe set (Phalanx Biotech Group, San Diego, CA) according to the manufacturer’s instructions. Data were uploaded to the Gene Expression Omnibus (GEO; accession number: GSE93194).

### Vector Construction, Transfection, and Lentivirus Transduction

Human full-length CCN3 cDNA (NM_002165) was obtained from GeneCards (Shanghai, China) and cloned into the pCDH lentiviral expression vector (System Biosciences, Palo Alto, CA, USA). The amplified fragment was inserted into the pCDH plasmid (between *Xba*I and *Eco*RI sites) using the In-Fusion® HD Cloning Kit (Takara, Tokyo, Japan). The target sequences of lentiviral shRNA expression plasmids PLKO.1 were CCCACCATCAAAGGAATATAA (Sh1), CGCACCAAGAAGTCACTCAAA (Sh2), and CACCAATAGGAACCGTCAATG (Sh3).

### RNA Extraction and Quantitative Reverse Transcription-Polymerase Chain Reaction (qRT-PCR)

Total RNA was extracted from HCC using TRIzol® reagent (Invitrogen, Carlsbad, CA, USA). The primers used for the amplification of human genes were 5′-CAGGCGGTAGAGGGAGATAA-3′ (forward) and 5′-TGGGCCACAGATCCACTTTTC-3′ (reverse).

### Tissue Microarrays Construction, Immunohistochemistry, Immunofluorescence, and Immuno-blotting

Tissue microarrays were constructed by Shanghai Biochip Co., Ltd. (Shanghai, China). Paraffin-embedded tissue sections (4 μm) were prepared according to established methods^30^. Immunohistochemistry, immunofluorescence, and immunoblotting were performed as previously described^12^. CCN3, OPN, and TF expression levels were evaluated in tissue microarrays and cell lines. MEK1/2 inhibitor U0126, NFκB inhibitor EVP4593, aspirin, and sorafenib were obtained from Selleckchem (Houston, TX, USA). Primary antibodies included CCN3, E-cadherin, TF, p-C-RAF, MEK, ERK1/2, and p-ERK1/2 (Abcam, Cambridge, MA, USA); OPN, thrombin, and C-RAF (Cell Signaling, Beverly, MA, USA); p-MEK (Epitomics, Burlingame, CA, USA); and Actin (Jackson Labs, Bar Harbor, ME, USA).

### Scoring of IHC staining results

IHC evaluation was determined independently by 2 pathologists without prior knowledge of the patients’ information. IHC is scored on a qualitative scale from 0 to 12, based on interpretation of staining intensity (negative staining = 0–2, weak staining = 3–6, moderate staining = 7–9, strong staining = 10–12) and the extent of stained area (<5% = 0, 6–25% = 1, 26–50% = 2, 51–75% = 3, å 75% = 4). The final score was determined by multiplying the intensity scores with the extent of positivity stained area, with the minimum score of 0 and a maximum score of 48. The mean percentage value of the 2 cores was considered representative of one tumor. The staining pattern was classified according to the final score as follows: negative (0–8), weak; (9–23): moderate (24–32); and strong (33–48). The final score of 24 was used as a cutoff value of high and low expression of CCN3

### Statistical Analysis

Statistical analyses were performed using SPSS 15.0 for Windows (SPSS, Inc., Chicago, IL, USA). Statistical significance was defined as p < 0.05.
